# Extracorporeal Cooling in Heat Stroke Induced Takotsubo Cardiomyopathy

**DOI:** 10.1155/crcc/9589641

**Published:** 2026-10-05

**Authors:** Maja Mačković, Nikolina Marić, Petar Pekić, Nikola Udiljak, Zrinka Planinić

**Affiliations:** ^1^ Intensive Care Unit Department of Internal Medicine Clinical Hospital Sveti Duh Zagreb Croatia kbsd.hr; ^2^ Department of Cardiovascular Diseases Department of Internal Medicine Clinical Hospital Sveti Duh Zagreb Croatia kbsd.hr

**Keywords:** cooling, heat stroke, hemodialysis, hyperthermia, takotsubo cardiomyopathy

## Abstract

Heat stroke is a life‐threatening condition characterized by elevated core body temperature (≥ 40°C) and central nervous system (CNS) dysfunction, resulting from body′s inability to adequately dissipate heat. It can lead to multiorgan failure, including the cardiovascular system. Takotsubo cardiomyopathy (TCM), also known as stress‐induced cardiomyopathy, is an acute syndrome of transient global or regional ventricular dysfunction, most commonly apical ballooning, mimicking acute coronary syndrome, typically occurring after severe physical or emotional stress. Although individually well‐described, the combination of severe heat stroke as a trigger for TCM is rare. This case report describes a female patient with severe heat stroke who developed TCM and, due to persistent hyperthermia unresponsive to conventional cooling methods, was successfully cooled using prolonged intermittent hemodiafiltration. It also highlights the importance of timely recognition of atypical complications such as TCM in patients with cardiovascular instability and demonstrates the potential role of hemodialysis as an effective cooling method in cases of refractory hyperthermia.

## 1. Introduction

Heat stroke represents a medical emergency with high rates of morbidity and mortality. It develops when the core body temperature rises above 40°C as a result of excessive heat gain or production, overwhelming the body′s thermoregulatory mechanisms, leading to organ dysfunction [1]. Two main forms include nonexertional heat stroke, occurring during periods of high ambient temperature affecting mostly vulnerable populations (elderly, chronically ill, and children), and exertional heat stroke, occurring in young, healthy individuals during intense physical activity in hot and humid conditions [2]. Pathophysiology involves a systemic inflammatory response, endothelial damage, and multiorgan failure affecting the brain, kidneys, liver, lungs, coagulation system, and cardiovascular system [3]. Cardiovascular complications of heat stroke can range from tachycardia and hypotension to arrhythmias, myocardial ischemia, and cardiogenic shock [4].

Takotsubo cardiomyopathy (TCM) is an acquired cardiomyopathy characterized by transient ventricular dysfunction, typically apical or midventricular “ballooning” of the left ventricle, in the absence of obstructive coronary artery disease [5]. Typical triggers include severe emotional or physical stress [6]. Proposed mechanisms include excessive catecholamine release with direct toxic effects on cardiomyocytes, microvascular dysfunction, and coronary artery spasm [7]. Diagnosis is based on the International Takotsubo Diagnostic Criteria (InterTAK Diagnostic Criteria) [8]. Although physical stress is a well‐known trigger for TCM, heat stroke as a primary and sole trigger is less frequently documented [9].

The main aspect of heat stroke management is rapid and effective reduction of body temperature below 39°C. Standard methods include surface cooling (removing clothing, spraying with water and fanning, and applying ice packs) and invasive methods (cooled intravenous fluids and lavage of body cavities with cold solutions) [10]. In severe cases with refractory hyperthermia and multiorgan failure, extracorporeal methods like extracorporeal membrane oxygenation (ECMO) or continuous renal replacement therapy (CRRT) can be utilized for cooling [11, 12]. The application of hemodialysis solely for cooling in patients with heat stroke is rarely reported but can be an effective strategy.

We present a case of a patient who suffered severe heat stroke, complicated by the development of TCM and whose persistent hyperthermia was successfully controlled using hemodialysis.

## 2. Case Presentation

A female patient in her 60s, with prior history of well controlled Type 2 diabetes and schizophrenia in remission, was admitted to the intensive care unit because of suspected heat stroke. The patient was found unconscious outdoors, lying on firm ground for an unknown period of time in high ambient temperature (> 30°C), without adequate fluid intake. Initial axillary temperature was 41.8°C, and cooling measures were promptly applied while being transferred to the hospital.

On admission, the patient was unconscious, unresponsive to painful stimuli, without muscular rigidity, hot, dry, and erythematous skin. Vital signs were as follows: rectal temperature 41°C, pulse 90/min, blood pressure 80/50 mmHg, respiratory rate 26/min. Following endotracheal intubation mechanical ventilation was commenced, and because of vasoplegic shock, vasoactive therapy with norepinephrine and vasopressin was initiated. Bedside FAST ultrasound protocol did not reveal any myocardial abnormalities.

Laboratory findings on admission revealed WBC 31.4 × 10^9^/L, Plt 106 × 10^9^/L, urea 13.5 mmol/L, creatinine 156 *μ*mol/L, sodium 140 mmol/L, potassium 6.1 mmol/L, CK 2068 U/L, AST 1254 U/L, ALT 1201 U/L, LDH 4170 U/L, CRP 1.5 mg/L, INR 1.81, aPTT prolonged (33.9), fibrinogen 2.9 g/L, arterial pH 7.29, pCO2 6.24 kPa, pO2 11.9 kPa (on FiO2 0.6), HCO3 20 mmol/L, lactate 2.8 mmol/L, hs Troponin I 1963 ng/mL. Electrocardiogram (ECG) showed sinus tachycardia without ST‐T abnormalities.

Extensive diagnostic workup including neuroimaging, electroencephalography, and clinical assessment excluded neurological condition as a cause of hyperpyrexia, predominantly neuroleptic malignant syndrome.

Aggressive cooling measures were immediately initiated, including removal of clothing, spraying skin with cold water and fanning, applying ice packs over major blood vessels, alongside the administration of cold balanced crystalloid solutions. Despite these measures, the rectal temperature dropped only to 39.9°C during the first several hours.

From the first out‐of‐hospital medical contact, continuous ECG monitoring was performed, presenting stable sinus rhythm without ventricular ectopy. Suddenly, 8 h after admission, the patient developed repetitive ventricular tachycardia without electrolyte abnormalities. ECG in between arrhythmia showed diffuse ST segment elevation with prolonged QTc (Figure 1). Given the elevation of cardiac markers (peak hsTnI 60951 ng/mL) and ECG changes, an urgent transthoracic echocardiogram (ECHO) was performed revealing significantly reduced left ventricular ejection fraction estimated at approximately 25%–30%, with akinetic aneurysmatically shaped apical segment. An urgent coronary angiography excluded obstructive coronary artery disease (Figure 2), confirming the diagnosis of TCM. Unfortunately, on invasive cardiologists′ discretion, ventriculography was not performed because it would offer little incremental diagnostic value as the InterTAK criteria permit a diagnosis without obligatory ventriculography. Moreover, it was decided to avoid excessive contrast burden with potential influence on already compromised renal function in hemodynamically unstable patient.

**Figure 1:**
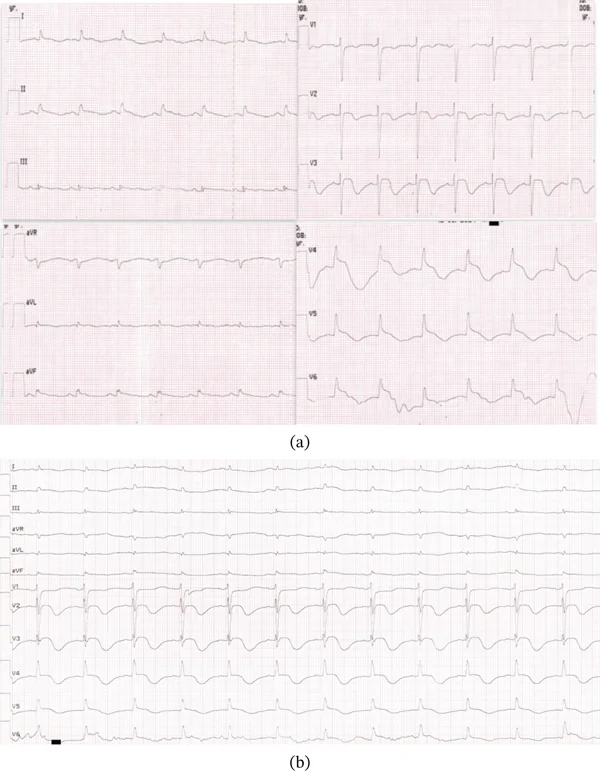
(a) ECG prior to coronary angiography with ST elevation, negative T‐waves in precordial leads, and QT prolongation. (b) ECG 2 h later (after coronary angiography), showing deep T‐wave inversion with significant QT prolongation; slight ST elevation persists in V3.

**Figure 2:**
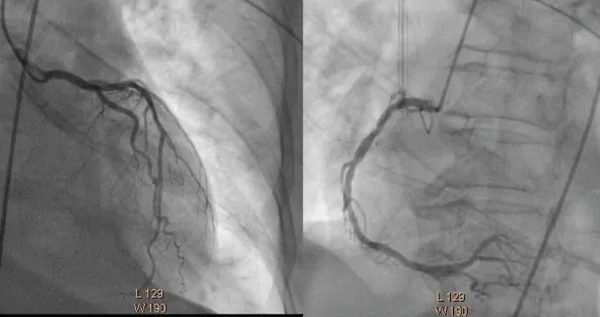
Coronary angiogram showing nonobstructive coronary artery disease.

Despite continuous cooling efforts, the body temperature ranged between 39°C and 40.1°C. Given the refractory hyperthermia which additionally aggravated hemodynamic instability and the risk of further neurological and cardiac damage, it was decided to apply extracorporeal cooling with a CRRT device. One 8‐h hemodiafiltration procedure using ST150 membrane (Baxter, Deerfield, Illinois, United States) was performed with a blood flow rate of 180 mL/min, room temperature (estimated 24°C–26°C) fluids and low effluent dose which successfully reduced her body temperature from 40.1°C to 37.8°C. During procedure, potassium was frequently monitored and remained between 3.9–4.1 mmol/L; by the end of the session, serum creatinine was 109 *μ*mol/L (initial value of 156 *μ*mol/L).

During the first week, the patient′s condition gradually improved but without significant neurological recovery. Prolonged mechanical ventilation due to concomitant ventilator‐associated pneumonia required percutaneous tracheostomy.

Serial control ECHOs showed mild recovery of left ventricular systolic function (LVEF estimated at approximately 35%–40%) without resolution of apical and midventricular hypokinesis despite standard heart failure therapy including bisoprolol 2.5 mg, empagliflozin 10 mg, eplerenone 25 mg, and perindopril 4 mg.

Eventually the patient was weaned from mechanical ventilation. Neurological recovery was slow, but subsequently, patient was conscious, oriented in communication, and able to perform simple motorical tasks requiring further intensive rehabilitation.

After 47 days of intensive care and rehabilitation, the patient was discharged from the hospital to a nursing home and subsequently lost to follow‐up.

The patient has been deidentified for this publication.

## 3. Discussion

This case report describes a complex clinical presentation of nonexertional heat stroke complicated by TCM where treatment of refractory hyperthermia included the application of hemodialysis.

Heat stroke is a condition that can lead to extensive organ damage, with cardiovascular system being particularly vulnerable. Extreme heat stress results in cutaneous vasodilation and increased cardiac output to enhance heat dissipation, which can provoke relative hypovolemia and hypotension. Furthermore, high temperatures directly damage cardiomyocytes, activate the sympathetic nervous system, and release catecholamines, potentially resulting in tachyarrhythmias, myocardial ischemia, and decreased contractility [4].

TCM is increasingly recognized as a potential complication of various conditions causing significant physical and emotional stress, sometimes intertwined or even without an identifiable trigger [6]. Heat stroke can be considered as a major physical stressor. The mechanisms by which heat stroke can precipitate TCM likely involve a massive release of catecholamines in response to the physiological shock caused by hyperthermia, hypotension and multiorgan failure, elevated catecholamine levels leading to multifocal myocardial damage, microvascular dysfunction, and dynamic left ventricular outflow tract obstruction, resulting in the typical “apical ballooning” appearance [7, 13]. Moreover, novel data suggest additional combination of hyperinflammation, endothelial cell damage, aberrant cardiac metabolism, and heat shock protein dysregulation as a precipitating mechanism [4]. Recently, studies have demonstrated that immune cell infiltration and secretion of both pro and anti‐inflammatory cytokines can contribute to TCM pathophysiology and outcome prediction [14]. Unfortunately, cytokine measurement (i.e.IL‐6) was not available in our institution at the time of presentation. Our patient presented with a classic TCM pattern on ECHO, elevation of cardiac markers, and ECG changes that can be seen in TCM. Exclusion of obstructive coronary artery disease confirmed the diagnosis. Other cases of TCM associated with heat stroke have been reported [9, 15], further supporting heat stroke as a potential trigger.

Management of heat stroke primarily focuses on rapid reduction of core body temperature as the patient prognosis is directly related to the degree and duration of hyperthermia. Standard methods of surface and invasive cooling are usually effective [10]. However, in severe cases with persistent hyperthermia and extensive multiorgan failure, conventional cooling may be insufficient. Inability to control hyperthermia exacerbates cellular damage, worsens metabolic acidosis, and contributes to hemodynamic instability [3]. In such situations, extracorporeal methods could offer an efficient tool for heat removal. ECMO can be used for both cooling and cardiopulmonary support in patients with severe cardiogenic shock and/or ARDS [11]. Renal replacement therapy (RRT) can also be adapted for cooling by using a cold dialysate [12, 16]. For hemodynamic stabilization, because of the similarities between heat stroke and sepsis, choice of the vasoactive agent should be as for the septic shock treatment, with norepinephrine being the first line therapy [17]. It cannot be excluded that the norepinephrine therapy as additional catecholaminergic load contributed to the development of acute cardiac dysfunction.

Although our patient did not develop oliguric acute kidney injury (AKI) and did not have a conventional indication for RRT, we decided to utilize extracorporeal treatment as a cooling method. The mechanism of cooling by hemodiafiltration is based on convective and diffusive heat transfer from the patient′s blood to the colder dialysate as blood passes through the dialyzer. By controlling the dialysate temperature, it is possible to achieve a significant reduction in body temperature. Several reports and studies have documented the effectiveness of extracorporeal therapy in controlling temperature in patients with various causes of hyperthermia [12, 18]. Its application in heat stroke, particularly when there is a simultaneous indication for dialysis due to AKI, represents an effective option.

Treatment of TCM is primarily supportive aimed at treating the underlying trigger and complications such as heart failure, arrhythmias, and thromboembolism [5, 6]. In our case, aggressive management of heat stroke and hemodynamic support likely contributed to the partial recovery of left ventricular function.

This case report describes a rare but possible association between severe heat stroke and TCM and demonstrates the successful application of RRT as a cooling method in a patient with refractory hyperthermia without concomitant oliguric AKI. TCM should be considered in heat stroke patients who develop cardiovascular instability or abnormalities on ECG/ECHO, and hemodialysis can be an effective means of temperature control. A similar cooling approach via RRT is mentioned in the literature as a valuable option in critical conditions [12].

## 4. Conclusion

Our case report illustrates the potentially life‐threatening combination of severe heat stroke, TCM, and refractory hyperthermia. The severe physical stress induced by heat can act as a trigger for TCM. In selected patients, RRT can be modified for cooling using cold solutions and represents an effective and life‐saving method for temperature control. Early diagnosis, aggressive cooling, and multiorgan support are crucial for improving outcomes in these complex clinical scenarios.

## Funding

No funding was received for this manuscript.

## Consent

No written consent has been obtained from the patient as there is no identifiable data included in this case report.

## Conflicts of Interest

The authors declare no conflicts of interest.

## Data Availability

The data that support the findings of this study are available from the corresponding author on reasonable request.
